# First report of uncommon mycobacteria in post LASIK keratitis: *Mycobacterium wolinskyi*

**DOI:** 10.1186/s12348-024-00438-6

**Published:** 2024-10-16

**Authors:** Sébastien van Delden, Hélène Buvelot, Giorgio Enrico Bravetti, Truong-Thanh Pham, Gabriele Thumann, Horace Massa

**Affiliations:** 1 Division of Ophthalmology, Department of Clinical Neurosciences, Geneva University Hospitals, Rue Alcide-Jentzer 22, Geneva, 1205 Switzerland; 2 Division of Infectious Diseases, Department of Medicine, Geneva University Hospitals, Geneva, Switzerland; 3 Faculty of Medicine, University of Geneva (UNIGE), Geneva, Switzerland

**Keywords:** *Mycobacterium wolinskyi*, Keratitis, Mycobacteria keratitis, LASIK, Refractive surgery, Cornea

## Abstract

Laser assisted in situ keratomileusis (LASIK) surgery is the leading and most performed refractive surgery nowadays. A possible complication of LASIK surgery is infectious keratitis which can lead to disastrous corneal damage and result in permanent loss of vision. LASIK procedures have become increasingly accessible, and the demand for refractive surgery has risen among patients, challenging the medical field to improve the prevention of post-operative infections. Nevertheless, a wide range of pathogens have been described as responsible for post-LASIK keratitis. However, non-tuberculous mycobacterial keratitis remains an infrequent occurrence and is poorly described in the literature. To the best of our knowledge, this is the first ever reported case of post-LASIK keratitis caused by *Mycobacterium wolinskyi.* We describe the clinical and microbial characteristics, leading to its challenging treatment choice.

## Introduction

Laser assisted in situ keratomileusis (LASIK) is currently the predominant method for refractive surgery due to its numerous advantages over other techniques in correcting ametropia [1]. It is well known for its very low complication rate, and overall patient satisfaction has been reported at over 95% of patients [2]. However, a rare but possibly devastating post-operative complication is infectious keratitis, which according to the largest series on this topic, has an incidence rate of 0.0046% [3] and 0.011% [4]. Microorganisms implicated in post-operative keratitis commonly comprise bacterial species, notably strains belonging to the *Staphylococcus* genus [5]. Furthermore, some studies reported case series involving non-tuberculous mycobacteria as one of the most common post-LASIK keratitis pathogens, accounting for up to 47% [6]. So far, six *Mycobacterium* species have been reported in the literature causing post-LASIK keratitis: *Mycobacterium chelonae*,* fortuitum*,* abscessus*,* mucogenicum*,* terrae* and *szulgai.* [7] The medical management of non-tuberculosis mycobacteria keratitis is challenging, and frequently remains unsatisfactory, primarily because of delayed diagnosis, insufficient drug penetration, and slow therapeutic response. Additionally, resistance to most conventional antibiotics and the development of resistant strains during prolonged treatment exacerbate the challenge of achieving favorable therapeutic outcomes [8].

To the best of our knowledge, we present the first ever reported case of corneal infection due to *Mycobacterium wolinskyi* following LASIK surgery. The patient provided written consent form; this work adheres to the principles outlined in the Declaration of Helsinki.

## Case description

A 49-year-old female patient was referred to our ophthalmologic clinic for an antibiotic-resistant left eye post-LASIK keratitis. The patient underwent the surgery 8 weeks earlier and presented first discomfort 12 days after. Prior to the referral, she had been treated initially as a conjunctivitis with topical tobramycin plus dexamethasone, and later, because of the degradation of her symptoms, as a bacterial keratitis, adding topical ciprofloxacin drops. At her arrival in our department, her treatment was tobramycin plus dexamethasone and hexamidin diisethionate drops, 3 times per day, artificial tears every 2 h and voriconazole 200 mg 2 times per day orally. She had no other ophthalmologic history and was only on thyroid replacement as medication.

**Fig. 1:**
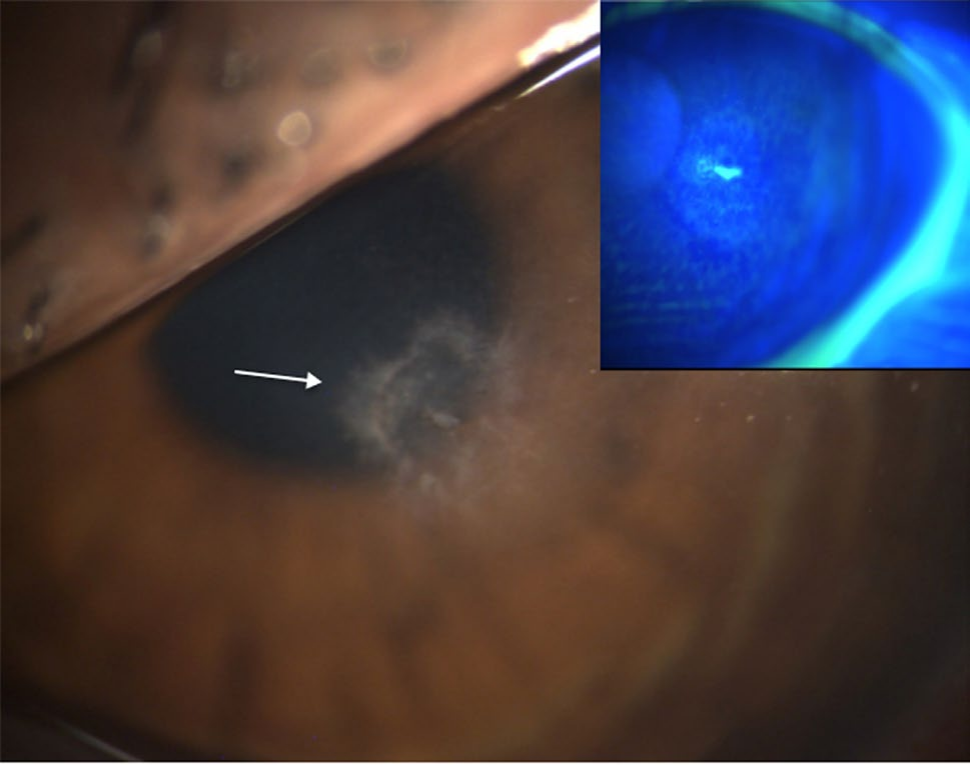
Slit lamp images of the left eye: white arrow pointing the mycobacterial keratitis’ ulcer. Upper right, the same image with fluorescein test revealing epithelium damage and the stromal impregnation

She presented to our clinic with conjunctival injection, blurred vision, foreign body sensation, photophobia and tearing of her left eye. The visual acuity was of 5/10 without correction and 8/10 with pinhole. Anamnestically, the initial visual acuity was 10/10. Intraocular pressure (IOP) was normal and corneal sensitivity was diminished but preserved in both eyes. Anterior segment examination of the right eye was within normal limits, with a calm post-LASIK interface. On the left eye, she presented with palpebral swelling, diffuse conjunctival hyperemia with chemosis, a paracentral corneal ulcer of 1 mm with anterior stromal infiltrate and an inflammatory anterior chamber (Tyndall 1+) (Fig. 1). Fundus examination in pharmacological mydriasis was unremarkable for both eyes. Anterior segment optical coherence tomography (OCT) revealed a ruptured epithelial flap in her left eye, associated with an anterior stromal infiltrate. Suspecting post-LASIK infectious keratitis, we stopped all treatment except the artificial tears for 24 h, and then performed multiple scrape tests at the ulcer’s base, without lifting the flap, for pathogen identification. The patient was hospitalized and initially received treatment of tobramycin drops and moxifloxacin hourly, day and night, natamycin drops 6x/day and scopolamine bromhydrate 2x/day. After 48 h, the night drops were replaced with tobramycin ointment.

**Fig. 2:**
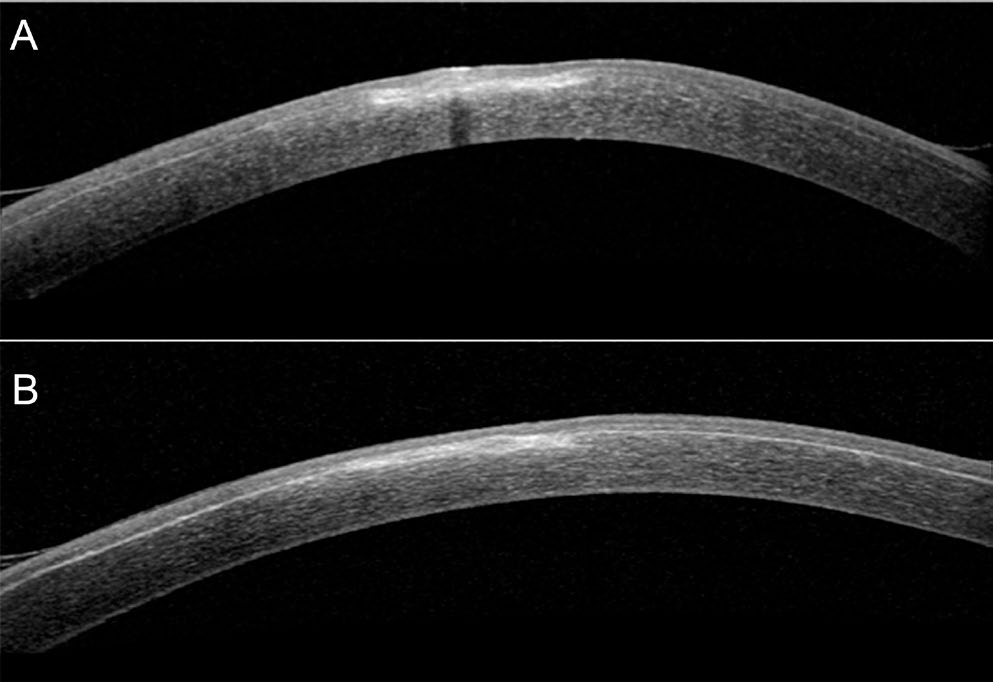
**A**) Anterior segment OCT revealing a foreign body highlighted by a shadow cone following his continuity. **B**) Anterior segment OCT post-surgical interface cleaning showing the disappearance of the shadow cone and the foreign body

The symptoms diminished progressively in the next days but did not resolve entirely. The cornea’s ulcer healed until no stain was visible under fluoresceine, but a stromal opacity remained with haze. The initial scrape revealed the presence of *Mycobacterium wolinskyi.* All tests remained negative for a co-infection with a fungus or Acantomoeba, as indicated by the confocal microscopy images performed during the workup. With the slightly better corneal visibility we recognized at slit lamp examination the presence, under the LASIK flap, of a stromal foreign body in the form of a microscopic whitish round material at the initial ulcer’s base. This was confirmed with an anterior segment optical coherence tomography (OCT) revealing a shadow cone following its continuity (Fig. 2A).

**Fig. 3:**
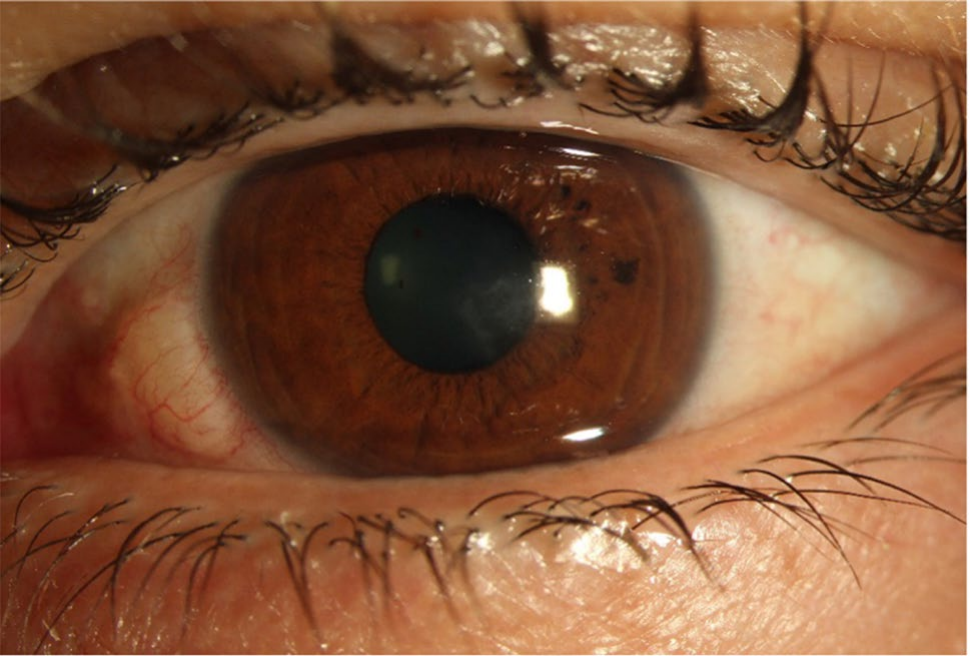
Slit lamp image of the cornea with a healthy epithelium and a sub epithelial scare after termination of all treatment

After providing an explanation and obtaining the patient’s approval, we performed surgical interface cleaning. While not lifting the flap, a gripper was used through the button pinhole created to swab the stromal foreign body. Gentle cleaning of the surroundings was then performed. The post-operative control anterior segment OCT no longer showed the shadow cone and confirmed the thorough removal of the foreign body (Fig. 2B). The patient was then treated with moxifloxacin drops, initially hourly, and then every 2 h after one week. After 11 days, she presented again to our clinic with an increase in pain and photophobia. The visual acuity was 2/10. The anterior segment examination showed an anterior stromal opacity with a slight central fluoresceine staining but no infiltrates. There was no satellite lesion, and the anterior chamber was calm. Due to the deterioration, we suspected an uncontrolled infection with *Mycobacterium wolinskyi* and sought assistance from the division of infectious diseases at our university hospital of Geneva. According to their recommendation and the possibility of hospital’s pharmacy to fabricate the eyedrops, we introduced in addition to moxifloxacin: amikacin 5% and erythromycin 0,5%. Each drop was instilled every 1.5 h during the day, resulting in a rotation of one antibiotic each 30 min. Tobramycin ointment was initiated for night treatment, and oral treatment of doxycycline 100 mg 2 times daily plus moxifloxacin 400 mg once daily was also prescribed. A new conjunctival swab was performed which did not give any positive results. A few days later, the antibiogram from the first initial swab revealed resistance of the mycobacterium to tobramycin and clarithromycin which motivated the discontinuation of tobramycin ointment at night and the daily drops of erythromycin. The symptoms diminished progressively, and corneal examination improved in the following weeks. After 12 weeks of treatment, oral treatment of doxycycline and moxifloxacin was stopped, and topical treatment of moxifloxacin and amikacin was diminished from one drop every 2 h to 6 times daily each. All drops were discontinued after 24 weeks of total treatment. At this point, after 6 months of treatment, the patient was asymptomatic, visual acuity improved to 10/10 and corneal examination showed a remaining paracentral stromal scar with no fluoresceine staining (Fig. 3).

## Discussion

This report shows, to the best of our knowledge, the first ever case of corneal infection due to *Mycobacterium wolinskyi* following LASIK surgery, and its challenging management.

As mycobacteria has been described in a high percentage of post-LASIK keratitis and their remarkable resistance to classic antibiotics resulting in poor outcomes [8], it is of utmost importance for ophthalmologist to recognize this pathogen and initiate the right treatment without delay. First described in 1999 [9], *Mycobacterium wolinskyi* is an atypical mycobacterium, fast growing, and part of group IV Runyon Classification of Nontuberculous Mycobacteria. In humans, it has been reported in less than 30 clinical cases in the literature, including bacteremia [10], peritonitis [11], infections associated with implants [12] /prostheses [13]–[14], and skin and soft tissue infections [15] often following surgical procedures [16]. It is commonly found throughout the environment in soil, dust, and water.

Due to the lack of literature on this specific post-LASIK keratitis, selecting the appropriate antibiotic treatment was particularly challenging. The collaboration with the infectious disease department and the literature review led us to the use of the previously cited drug combination. This was decided according to the reports in the literature on other non-tuberculous mycobacterium which recommends the triple association of topical amikacin (50 mg/L), clarithromycin (10 mg/L), and fourth-generation fluoroquinolones. However, the use of steroids is prohibited. Systemic antibiotics as clarithromycin and doxycycline are also considered an addition in recalcitrant cases. There are no clear guidelines for treatment duration which varies from 6 weeks to 6 months [17]. 

Regarding these recommendations we had to switch the oral clarithromycin to moxifloxacin 400 mg once daily because of our pathogen particular antibiogram resistance. For the same reason, and due to its unavailability in droplet form in the hospital’s pharmacy, we substituted the topical clarithromycin with erythromycin 0.5%.

In this case, the patient had two major risk factors: first the LASIK surgical procedure, but also and most importantly here, the microscopic stromal foreign body. The corneal spectral-domain OCT enabled the location and diagnosis thanks to the shadow cone highlighted. Furthermore, this case report pleads in favor of the removal of corneal foreign body in an infectious setting. The balance here in the surgical context resides in a thorough and meticulous interface cleaning through the created pinhole button and not in lifting completely the flap or its resection. The proposed management resulted in an excellent final visual acuity and the preservation of the LASIK surgical work. An alternative would have been a partial or complete flap lifting to clean the area of infection. Nevertheless, in this context, it was assumed that the keratitis induced a strong inflammatory reaction. Thus, flap’s stromal junction must have been made very adherent, limiting by this way the risk of epithelial ingrowth, which is a classical complication in buttonhole flap perforation. Therefore, direct treatment through the ulcer was chosen.

Post-LASIK keratitis and mycobacterial infection are both rare events. However, the abovementioned high rate of this atypical pathogen in this setting is surprisingly high which makes it an important concern for ophthalmologist to be aware of. The recognition of a foreign body on anterior segment OCT, its proper removal, interface cleaning without lifting the flap, and the antibiotic combination associated with a long duration treatment of 6 months were all determining factors in the outcome.

## Data Availability

No datasets were generated or analysed during the current study.
